# CT Texture Analysis of Pulmonary Neuroendocrine Tumors—Associations with Tumor Grading and Proliferation

**DOI:** 10.3390/jcm10235571

**Published:** 2021-11-26

**Authors:** Hans-Jonas Meyer, Jakob Leonhardi, Anne Kathrin Höhn, Johanna Pappisch, Hubert Wirtz, Timm Denecke, Armin Frille

**Affiliations:** 1 Department of Diagnostic and Interventional Radiology, University of Leipzig, 04103 Leipzig, Germany; jakob.leonhardi@medizin.uni-leipzig.de (J.L.); timm.denecke@medizin.uni-leipzig.de (T.D.); 2 Department of Pathology, University of Leipzig, 04103 Leipzig, Germany; annekathrin.hoehn@medizin.uni-leipzig.de; 3 Department of Respiratory Medicine, University of Leipzig, 04103 Leipzig, Germany; johanna.pappisch@medizin.uni-leipzig.de (J.P.); hubert.wirtz@medizin.uni-leipzig.de (H.W.); armin.frille@medizin.uni-leipzig.de (A.F.); 4 Integrated Research and Treatment Center (IFB) Adiposity Diseases, University Medical Center Leipzig, 04103 Leipzig, Germany

**Keywords:** computed tomography, texture analysis, neuroendocrine tumor

## Abstract

Texture analysis derived from computed tomography (CT) might be able to provide clinically relevant imaging biomarkers and might be associated with histopathological features in tumors. The present study sought to elucidate the possible associations between texture features derived from CT images with proliferation index Ki-67 and grading in pulmonary neuroendocrine tumors. Overall, 38 patients (*n* = 22 females, 58%) with a mean age of 60.8 ± 15.2 years were included into this retrospective study. The texture analysis was performed using the free available Mazda software. All tumors were histopathologically confirmed. In discrimination analysis, “S(1,1)SumEntrp” was significantly different between typical and atypical carcinoids (mean 1.74 ± 0.11 versus 1.79 ± 0.14, *p* = 0.007). The correlation analysis revealed a moderate positive association between Ki-67 index with the first order parameter kurtosis (*r* = 0.66, *p* = 0.001). Several other texture features were associated with the Ki-67 index, the highest correlation coefficient showed “S(4,4)InvDfMom” (*r* = 0.59, *p* = 0.004). Several texture features derived from CT were associated with the proliferation index Ki-67 and might therefore be a valuable novel biomarker in pulmonary neuroendocrine tumors. “Sumentrp” might be a promising parameter to aid in the discrimination between typical and atypical carcinoids.

## 1. Introduction

Texture analysis is an emergent imaging analysis that quantifies radiological images and thereby can provide novel imaging biomarkers [1,2,3]. Every radiological modality is principally applicable for this analysis. Images derived from computed tomography (CT) are readily analyzed because of its robust imaging acquisition and high availability [1,2,3].

Texture analysis is divided into first order features, or more commonly termed histogram features, which are calculated by issuing the image features into a histogram without concern of their spatial relationships [1]. Hence, these parameters, comprising skewness, kurtosis, and entropy, can reflect different histopathological parameters in several tumor entities [3,4,5,6,7,8]. Moreover, it was identified that these features aid to predict treatment response and also help discriminate between different tumor types [3,9,10]. Altogether, these parameters have been shown to be reliable enough for clinical translation and their possible correlations with histopathology are being more and more elucidated [3].

The second important group of features is the second order statistics, which are typically called texture features [1]. They describe spatial relationships between voxels with similar gray levels within a lesion. They provide a measure of intralesional heterogeneity and might, therefore, be even more useful than the first order features. Commonly applied techniques are the Gray Level Co-occurrence Matrix, the Gray Level Run-Length Matrix, and the Gray Tone Difference Matrix [1]. For the latter, higher-order statistics impose filter grids on an image to extract repetitive or non-repetitive patterns [1].

In a first groundbreaking study based upon 1019 lung and head and neck cancer patients, texture analysis derived from CT images was capable to stratify patients according to their prognosis, thus generating a novel phenotypical characterization of tumors derived from CT images [11]. Since then, the literature regarding CT texture analysis has been expanding throughout numerous radiological fields.

Pulmonary neuroendocrine tumors (pNET) are discriminated into typical (TC) and atypical (AC) carcinoids [12,13]. Currently, pNET account for only 1–2% of all invasive lung malignancies. The ratio between TC and AC is about 8–10:1, which implies that AC is the most uncommon type of pNET [12].

The discrimination between these tumor types is crucial as TCs are metastatic in up to 15% of cases, usually to regional lymph nodes, whereas ACs are regionally or distantly metastatic in up to one half [12].

In oncology, the Ki-67 labeling of tumor cells is a clinically widely applied immunohistochemically parameter [14,15]. It reflects the proliferation rate of tumors and is, thereby, a marker for malignancy. For pNET, Ki-67 staining is a key parameter for grading purposes [12]. The main role of Ki-67 in pNET is to help distinguish carcinoid tumors (NETs) from large-cell neuroendocrine carcinoma (LCNEC) and small-cell lung cancer (SCLC), especially in small biopsies with crush artefacts, where carcinoids can be mistaken for SCLC [16]. A mitosis count of 2 per 2 mm² is used to discriminate between TC and AT [12]. Beyond that, necrosis is a main histological feature, which is used for the diagnosis of atypical carcinoid [12].

The principal hypothesis of this work was that texture analysis might be able to reflect these distinctive histopathological features and represent a reliable tool in clinical routine to characterize these tumors in a non-invasive manner (e.g., during initial staging). Therefore, the aim of the present study was to employ texture analysis derived from CT images to elucidate possible associations with proliferation index (Ki-67) and grading in pNET.

## 2. Material and Methods

### 2.1. Study Design

Standardized clinical and pathological (histology, tumor stage) and survival data from patients with lung cancer (ICD-10 C34.*) who were diagnosed at the University Hospital Leipzig were derived from the regional, clinical cancer registry of Leipzig (Regionales Klinisches Krebsregister Leipzig, Philipp-Rosenthal-Straße 27 b, Haus R, Leipzig, Germany).

The in-house radiological database was retrospectively screened for those cases selected between the years 2001 and 2018 for pulmonary neuroendocrine tumors.

Inclusion criteria for pNET consisted of available presurgical or prebiopsy CT data and corresponding comprehensive histopathological analysis of the tumors including Ki-67 index calculation. Exclusion criteria comprised all lung cancer cases, whose histology was not classified pNET, artifacts on CT images, and cases without histopathological analysis.

### 2.2. Image Analysis

CT imaging was performed in a clinical setting with a 16 slices CT scanner (Big Bore 16, Philips, Amsterdam, the Netherlands) or with a 256 slices CT scanner (iCT, Philips). In 18 cases (47% of patients), 90 mL of iodinated intravenous contrast medium was given at a rate of 1.5 to 3.5 mL/s by a power injector (Medtron GmbH, Saarbrücken, Germany), with a scan delay of 30 s after onset of injection. Typical imaging parameters were 120 kVp, 150 to 300 mAs, and a slice thickness of 1 mm. The anatomical localization of the pNET was reported according to the lung lobe. The diameter of the tumor was estimated on the largest slide of the tumor. The localization was defined as peripheral (2 cm or less distance to the visceral pleura) or central (2 cm or more distance to the visceral pleura).

### 2.3. Texture Analysis

**Figure 1:**
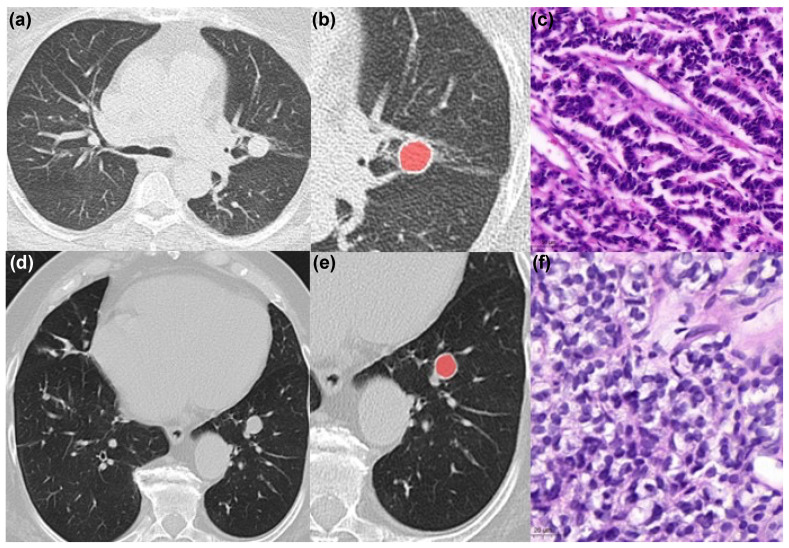
Representative cases of the patient sample with a typical carcinoid (**a**–**c**) and atypical carcinoid (**d**–**f**). Typical carcinoid is located centrally in the left upper lobe. The corresponding Ki-67 index was 1%. The region of interest of this tumor is drawn within the tumor boundaries (**b**). Histopathological image of the typical carcinoid (hematoxylin eosin stain) is shown in panel (**c**). Another representative case of the patient sample with an atypical carcinoid located centrally in the left lower lobe (**d**–**f**). The corresponding Ki-67 index was 5%. The region of interest of this tumor is drawn (**e**). Histopathological image of the atypical carcinoid (hematoxylin eosin stain) is shown in panel (**f**).

CT images were transformed into DICOM format and further processed with the free available texture analysis software MaZda (version 4.7, available at http://www.eletel.p.lodz.pl/mazda/, accessed on 1 February 2021) [17,18]. A polygonal region of interest (ROI) was placed on the largest, representative slide within the boundary of the tumor on one axial slide in a blinded manner to the histopathology results. Images were analyzed in 1 mm lung kernel reconstructed slices. For each ROI, gray-level normalization was performed, using the limitation of dynamics to μ ± 3 SD (μ gray level mean, SD standard deviation) to minimize the influence of contrast and brightness variation, as it was performed in previous studies [8,19]. The extracted features were as follows: gray-level histogram co-occurrence matrix (angular second moment, contrast, correlation, entropy, sum entropy, sum of squares, sum average, sum variance, inverse difference moment, difference entropy, difference variance (for four directions), and run-length matrix (run-length non-uniformity, gray-level non-uniformity, long run emphasis, short run emphasis, and fraction of image in runs)), absolute gradient, autoregressive model (theta 1 to 4, sigma), and wavelet transform. Altogether, 279 texture features were retrieved from every tumor. Figure 1 displays 2 representative cases of the patient sample.

### 2.4. Histopathology Analysis

All neuroendocrine tumors were histopathologically analyzed blinded to the imaging results. The proliferation index was estimated on Ki-67 antigen-stained specimens using MIB-1 monoclonal antibody (DakoCytomation, Glostrup, Denmark). The Ki-67 index was calculated at an area with the highest number of stained nucleic per 100 cells.

### 2.5. Statistical Analysis

Statistical analysis was performed using GraphPad Prism 5 (GraphPad Software, La Jolla, CA, USA). Collected data were evaluated by means or medians of descriptive statistics (absolute and relative frequencies). Spearman’s correlation coefficient (r) was used to analyze associations between texture features and Ki-67 index. Differences between typical and atypical carcinoids were investigated by two-tailed Mann–Whitney test. Receiver operating characteristics (ROC) analyses with calculation of the area under the curve (AUC) were performed to test for discrimination purposes. In all instances, *p*-values < 0.05 were taken to indicate statistical significance.

## 3. Results

Overall, 38 patients (*n* = 22 females, 58%) with a mean age of 60.8 ± 15.2 years with histopathologically proven primary NET of the lung were included in the present analysis.

An overview of the descriptive statistics of the included patients is given by Table 1.

Of all carcinoid tumors, 25 (66%) were TC and 13 (34%) were AC. The tumor localizations were as follows: the left lower lobe (*n* = 11, 29%), the right lower lobe (*n* = 9, 24%), the left upper lobe (*n* = 7, 18%), the right upper lobe (*n* = 4, 11%), endobronchial (*n* = 4, 10%), and in the middle lobe (*n* = 3, 8%). A total of 21 of the tumors (55%) were located centrally, while 17 (45%) were located peripherally.

### 3.1. Correlation and Discrimination Analysis with Grading

In Spearman’s correlation analysis, no texture parameter showed a statistically significant association with grading.

**Figure 2:**
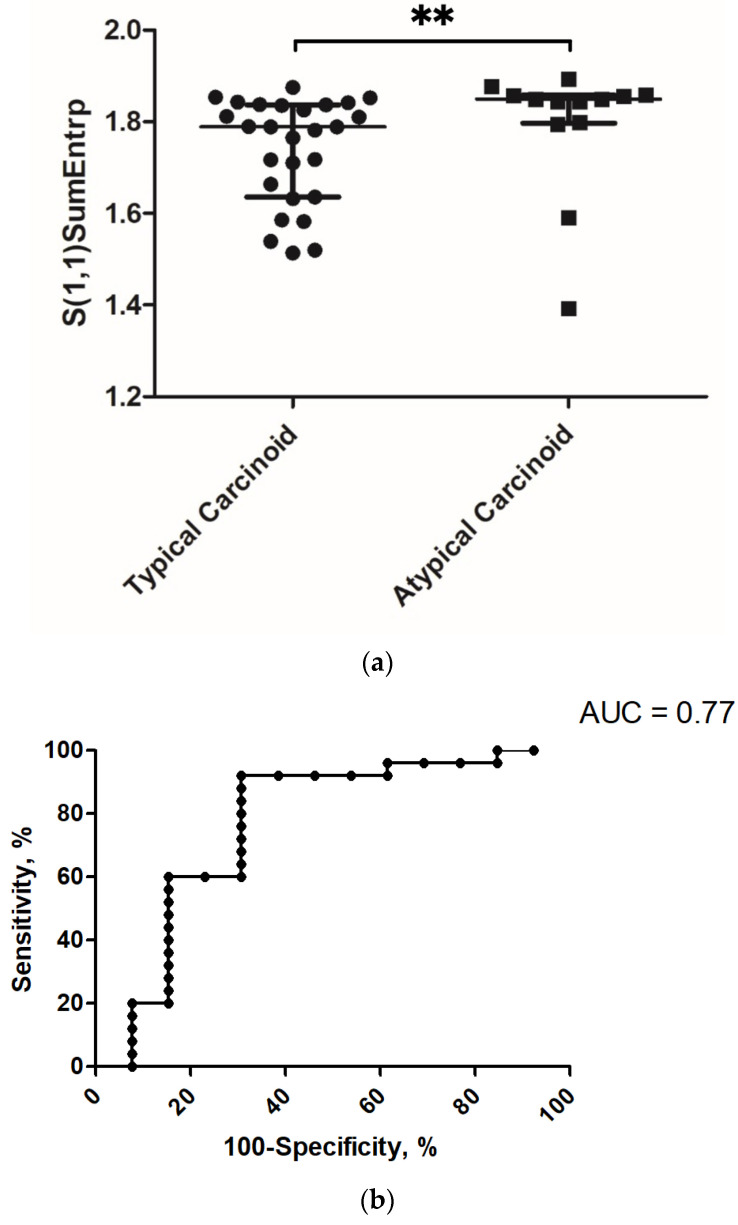
(**a**) Discrimination analysis between typical and atypical carcinoids. The parameter “S(1,1)SumEntrp” reached statistical significance (Mann–Whitney test) mean 1.74 ± 0.11 versus 1.79 ± 0.14, *p* = 0.007, ** *p* < 0.01). (**b**) ROC graph for the discrimination analysis between typical and atypical carcinoids with “S(1,1)SumEntrp) employing the threshold of 1.84. AUC of 0.77 showed a sensitivity of 0.92 and a specificity of 0.69.

In discrimination analysis, the following parameters were statistically different between TC and AC: “S(1,1)SumEntrp” (mean 1.74 ± 0.11 versus 1.79 ± 0.14, *p* = 0.007, Figure 2a), “S(2,0)SumEntrp” (mean 1.71 ± 0.11 versus 1.77 ± 0.14, *p* = 0.048), “S(2,2)SumEntrp” (mean 1.67 ± 0.10 versus 1.73 ± 0.13, *p* = 0.028), “S(3,0)SumEntrp (mean 1.67 ± 0.10 versus 1.73 ± 0.13, *p* = 0.032)”, “S(4,0)SumEntrp” (mean 1.65 ± 0.11 versus 1.72 ± 0.14, *p* = 0.021), and “S(5,0)SumEntrp” (mean 1.65 ± 0.11 versus 1.70 ± 0.14, *p* = 0.035).

“S(1,1)SumEntrp” was further investigated by ROC analysis. It achieved an area under the curve of 0.77 with a sensitivity of 0.92 and a specificity of 0.69 using a threshold value of 1.84. Figure 2b displays the corresponding graph.

### 3.2. Correlation Analysis with Ki-67 Index

In 22 patients (58% of all cases), the Ki-67 index was available for analysis. The Ki-67 index had a median of 1.5% and a range from 1 to 60%.

The correlation analysis revealed a positive association with the first order parameter kurtosis (*r* = 0.66, *p* = 0.001,).

**Figure 3:**
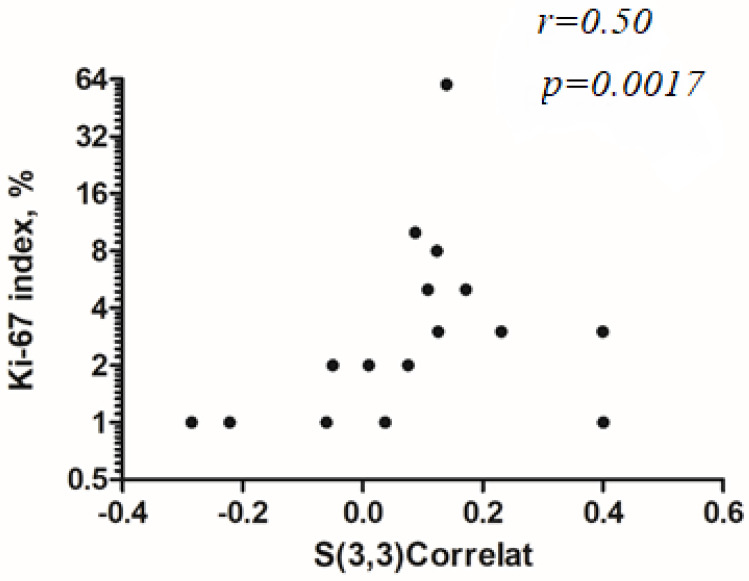
Spearman’s correlation analysis between Ki-67 index and “S(3,3)Correlat” (*r* = 0.50, *p* = 0.017). The Ki-67 axis is displayed on a log 2 scale.

**Figure 4:**
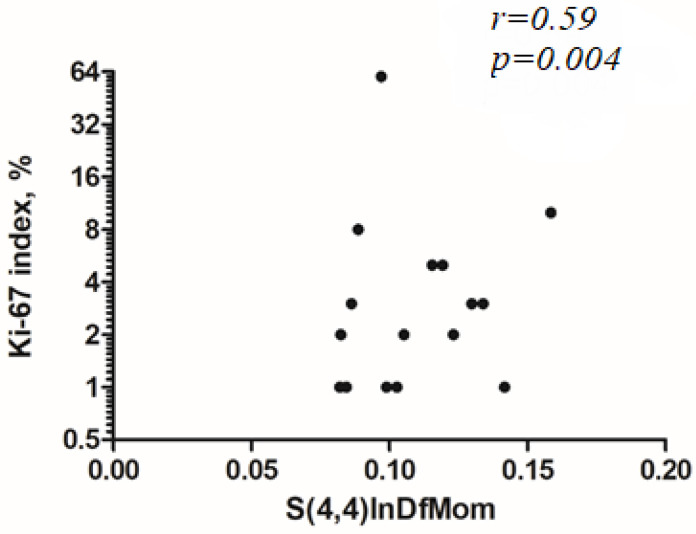
Spearman’s correlation analysis between Ki-67 index with “S(4,4)InvDfMom” (*r* = 0.59, *p* = 0.004). The Ki-67 axis is displayed on a log 2 scale.

For second order features, “S(3,3)Contrast” reached statistical significance with (*r* = −0.47, *p* = 0.027), “S(3,3)Correlat” with (*r* = 0.50, *p* = 0.017, Figure 3), “S(3,3)InvDfMom” (*r* = 0.48, *p* = 0.024), “S(4,4)InvDfMom” (*r* = 0.59, *p* = 0.004, Figure 4), and “S(0,5)Correlat” (*r* = 0.45, *p* = 0.034). The other investigated texture features did not reach statistical significance.

### 3.3. Discrimination Analysis for Ki-67 Index

A clinically recommended threshold of 2% for pNET was used for discrimination analysis.

In ROC analysis, Kurtosis reached an AUC of 0.91, followed by “S(3,3)Correlat” with 0.87, “S(3,3)Contrast” with 0.84, “S(4,4)InDfMom” with 0.77, and “S(3,3)InvDfMom” with 0.75.

**Figure 5:**
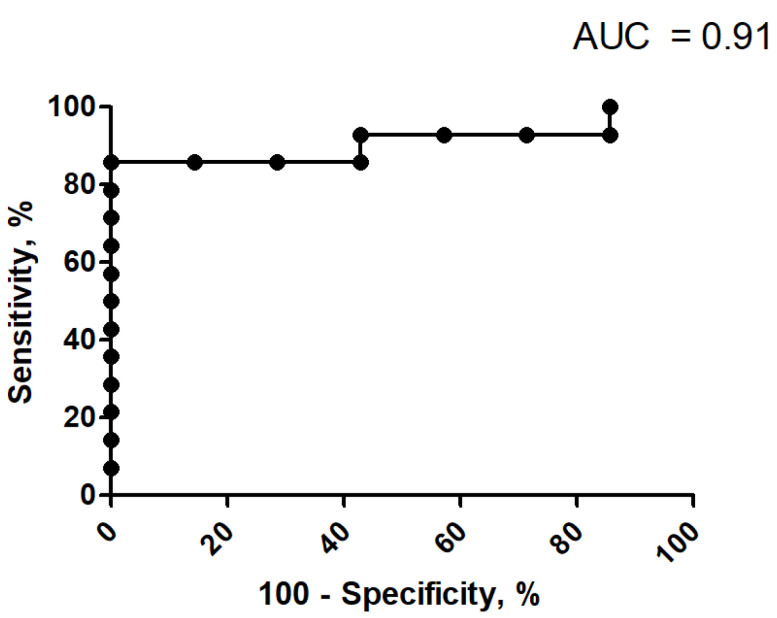
ROC graph for the discrimination analysis of pNET with a Ki-67 threshold of 2%. Kurtosis reached an AUC of 0.91 with a sensitivity and specificity of 0.86 employing the threshold value of 0.30.

Kurtosis reached a sensitivity and specificity of 0.86 employing the threshold value of 0.30. The corresponding graph is shown in Figure 5.

### 3.4. Correlation Analysis between Localization and Lesion Size

In terms of tumor localization, central and peripheral lesions showed significant differences regarding the following texture parameters: Mean (mean 205.5 ± 10.9 versus 214 ± 14.3, *p* = 0.043), Perc, 01% (mean 183.2 ± 13.6 versus 194.8 ± 10.9, *p* = 0.01), and Perc, 10% (mean 194.1 ± 9.9 versus 203.3 ± 12.1, *p* = 0.02).

In Spearman‘s correlation analysis, the following texture parameters showed statistically significant associations with the lesion’s size: “S(0,5)SumVarnc” (*r* = 0.39, *p* = 0.014), “Horzl_RLNonUni” (*r* = 0.78, *p* ≤ 0.001), “Horzl_GLevNonU” (*r* = 0.86, *p* ≤ 0.001), “Vertl_RLNonUni” (*r* = 0.75, *p* ≤ 0.001), “Vertl_GLevNonU” (*r* = 0.84, *p* ≤ 0.001), “45dgr_RLnonUni” (*r* = 0.77 *p* ≤ 0.001), “45dgr_GLevNonU” (*r* = 0.84, *p* ≤ 0.001), “135dr_GLevNonU” (*r* = 0.83, *p* ≤ 0.001), “Teta 2” (*r* = 0.65, *p* ≤ 0.001), “Sigma” (*r* = 0.47, *p* = 0.002), and “WavEnLL_s-3” (*r* = 0.47, *p* = 0.002).

## 4. Discussion

The present study identified statistically significant associations between texture features derived from CT images and tumor cell proliferation (Ki-67) in pNET. Another key finding is that texture features might help discriminate TC from AC. These findings could be of great importance in clinical routine as they might allow a non-invasive tumor characterization by applying CT imaging. Ki-67 labeling is useful in biopsy for distinguishing TC and AC from SCLC cytology, but it does not reliably distinguish TC from AC in any material [12].

The morphological analysis of CT imaging features of a pNET are often described as nonspecific and may have similar appearances to adenocarcinoma of the lung [20,21]. The most common appearance is a round or ovoid shape peripheral lung nodule with smooth or lobular margins [21]. They tend to grow slower compared to a lung carcinoma.

The biological aggressiveness of pNET differs significantly according to grading [12]. If radiologists are able to predict the underlying pathological tumor type by CT imaging, this will improve diagnostic power in the clinical routine and will potentially reduce invasive procedures, such as presurgical biopsies. This is moreover of note as more than 40% of the cases may be incidentally detectable by imaging [21], which imposes the main clinical question concerning the possible biological behavior of newly identified tumors. This is also of clinical interest as treatment decisions depend on the histological type and AC show worse prognosis compared to TC.

The present results suggest that “Sumentrp” could be a promising parameter, since it might help discriminate TC from AC. An advanced entropy-based parameter, “Sumentrp”, is well known for its ability to quantify the heterogeneity of tumors, being also associated with underlying histopathological features [3]. CT heterogeneity of tumors are assumed to be based on microstructural characteristics. As mentioned, necrosis is a key feature of AC, which might result in differences in the texture features. Necrotic areas might exhibit a more irregular and inhomogeneous nature compared to non-necrotic areas.

Interestingly, these findings are supported by another recent study investigating CT texture analysis to discriminate benign from malignant solitary pulmonary nodules [22]. Therein, entropy parameters were most adept in discriminating nodules according to their dignity [22].

In another study, texture analysis with a radiomics approach predicted CD8+ lymphocytes in lung cancer patients in a multi-center design undergoing immunotherapy [23]. Beyond that, the radiomics signature identified was significantly associated with the overall survival (Hazard ratio 0.58) [23].

Preliminary studies with promising results further investigated direct correlations between histopathology and CT texture in lung cancer entities [24,25]. These studies highly encouraged the possibility that modern imaging analysis techniques can reflect prognostic relevant histopathological features in lung tumors, which might result in a different clinical diagnostic work-up of lung cancer patients.

In this study, several CT texture parameters showed correlations with the proliferation index (Ki-67). The proliferation index is an essential feature for grading purposes in pNET [12,13,14]. In fact, the discrimination between carcinoid tumors and LCNEC or SCLC currently depends on the proliferation index (Ki-67) [12]. As shown, the parameter Kurtosis reached a high accuracy to predict the correct Ki-67 level.

A recent study investigated whether morphological CT features and [^18^F]fluorodexoyglucose-positron emission tomography (FDG-PET) were associated with the Ki-67 index [26]. Therein, the presence of irregular margins, bronchial obstruction, lymph nodes, and metastases were significantly correlated with a higher grade of Ki-67 index [26]. Moreover, as expected, the Ki-67 index was positively associated with the standardized uptake values (SUV) derived from FDG-PET linking glucose metabolism to proliferation activity. The present study adds to this topic that the possibility of CT texture analysis provided another measure to reflect Ki-67 index with imaging modalities applied in clinical routine. Yet, further studies, at best in a multicentric design, are needed to validate these reported preliminary results.

The importance of the prediction of the Ki-67 index can be underlined by a recent study on 176 carcinoid tumors with histopathology examination [14]. The authors demonstrated that the Ki-67 index was the only significant predictor of tumor recurrence in a multivariate analysis among all pulmonary carcinoid tumors and within TC tumors alone, whereas the histologic patterns or lymphovascular invasion status were not of predictive value [14].

In another recent study, the benefit of radiomics derived from FDG-PET were investigated in 44 patients with pNET [27]. Interestingly, the authors could not identify a significant benefit of the radiomics parameters in addition to the conventional FDG-PET parameters.

In another study of liver metastasis derived from pancreatic and non-pancreatic NET, there were associations between texture features and grading as well as with mortality [28].

A further study utilizing MaZda software, as performed in the present study, used texture analysis to predict Ki-67 in non-small-cell lung cancer patients [29]. The authors could establish a classifier with a resulting area under the curve of 0.78, which seems to be a reliable non-invasive method to predict Ki-67 status in these lung cancer patients [28]. For other tumor entities, there were some weak correlations reported between CT texture analysis and Ki-67 index in head and neck squamous cell carcinoma [7].

In hepatocellular carcinoma, several texture features, including entropy, were associated with the Ki-67 index and were able to discriminate between patients with low and high Ki-67 indices [30]. There is growing evidence that the Ki-67 index can be predicted non-invasively by modern imaging based on CT images.

Yet, it should be emphasized that other immunohistochemical parameters may be investigated and correlated with CT texture analysis. Traditional markers for neuroendocrine tumors comprise synaptophysin, chromogranin A, and CD56 (NCAM1). Another interesting marker is insulinoma-associated protein 1 (INSM1), which reflects true neuroendocrine differentiation [31]. For future studies, it would be of interest, whether these histopathological parameters could also be reflected by quantitative imaging analysis, including the biological heterogeneity of pNET [32].

The stability of texture features was investigated in previous studies [33,34,35]. In addition to different scanner techniques and image reconstruction methods, the lesion size should be considered as a possible factor influencing texture features. As shown, there are slight differences in the texture features in small regions of interests compared to large ones [34]. In general, however, CT-derived features are considered more reliable compared with magnetic resonance imaging derived texture features [34]. The results presented in here may also suggest that several texture features were associated with the lesion size, some of them even with strong correlation coefficients. Few texture features showed significant difference between centrally and peripherally located tumors. However, these features were not associated with proliferation index or grading but could potentially influence the texture features in a relevant manner.

Concerning contrast media application, texture features might slightly differ between native CT scans and contrast-enhanced CT scans. A recent study employed texture analysis in native CT scans derived from PET-CT to discriminate peripheral lung cancer and inflammatory pseudotumor with promising results [36]. Moreover, reconstruction algorithms of the CT images seem to influence radiomics features [37].

There are several limitations of the present study to address. First, this retrospective study shows possible known inherent bias. Second, the patient sample is rather small based upon the low prevalence of pNET among lung tumors. Moreover, the present study was a single-center analysis, which nevertheless was the first patient sample reported to date. Third, only 60% of patients had a Ki-67 index available, which further reduced the size of the patient sample. Fourth, the Ki-67 index was evaluated on a presurgical biopsy and might therefore not be fully representative of the whole tumor, whereas the CT texture analysis was performed on the biggest slide of the tumor, which might lead to spatial incongruencies. Fifth, we used two different CT scanners of the same vendor. It has to be acknowledged that the use of two different scanners might have caused some differences in the texture features analysis, which we could not account for at the moment. Other factors, such as contrast media application, lesion size, and localization, could influence the texture features analysis as well.

In conclusion, several texture features derived from CT were associated with the proliferation index based on Ki-67 labeling and might therefore be potentially valuable and novel biomarkers in pNET. “Sumentrp” might be a promising parameter that helps discriminate TC from AC. “Kurtosis” could non-invasively predict the Ki-67 level of 2% with a high accuracy. The added value of CT texture analysis should be investigated in further studies and possibly translated into clinical routine.

## Figures and Tables

**Table 1:** Localization and size of the pulmonary neuroendocrine tumors.

	Typical Carcinoid (%)	Atypical Carcinoid (%)	Total Amount (%)
Total amount	25 (66)	13 (34)	38 (100)
Lung lobe			
right upper lobe	0	4	4 (11)
middle lobe	3	0	3 (8)
right lower lobe	7	2	9 (24)
left upper lobe	5	2	7 (18)
left lower lobe	6	5	11 (29)
endobronchial	4	0	4 (10)
Localization			
central (>2 cm distance from pleura)	14	7	21 (55)
peripheral (≤2 cm distance from pleura)	11	6	17 (45)
**Size** in mm (mean ± SD) range	20.2 ± 16.3 5.5–74.3	23.4 ± 19.4 10–84.1	

## Data Availability

The original data and the statistical analyses can be obtained from the corresponding author upon request.

## Abbreviations

CT—Computed tomography, NET—neuroendocrine tumor, TC—typical carcinoid, AC—atypical carcinoid.
